# Case report: 18F-FDG PET confirmed pupil-sparing third nerve palsy heralding aseptic cavernous sinus embolism in patient with chest malignancy

**DOI:** 10.3389/fsurg.2022.893651

**Published:** 2022-08-31

**Authors:** Jianmei Xiong, Huanbo Liu, Jianyong Li, Jiajia Hou, Fang Cui

**Affiliations:** ^1^ Department of Neurology, Hainan Hospital of Chinese PLA General Hospital, Hainan, China; ^2^ Department of Cardiology, The Second Navy Hospital of Southern Theater Command of PLA, Hainan, China

**Keywords:** cancer embolus, diplopia, lung, cavernous sinus embolism, 18F-FDG PET

## Abstract

Classical cavernous sinus embolism is a rare clinical finding, presented most commonly by complaints of headache, diplopia, visual field defects, facial pain, and progressive neurological deficits. Many patients exhibit symptoms of III, IV, and VI nerve palsies. We hereby report a rare case of aseptic cavernous sinus embolism developed in a 75-year-old male with primary lung cancer who presented with binocular diplopia due to unilateral third and sixth cranial nerve palsies with pupil-sparing. The possibility of cavernous sinus cancer embolus should be considered if the routine examination excluded metastases, infiltration, carcinomatous meningitis, or the paraneoplastic process. ^18^F-FDG PET imaging may provide a promising diagnostic modality for the diagnosis of cancer embolus.

## Introduction

Unilateral ophthalmoplegia associated with pain, paresthesias, and numbness in the distribution of the V1 and V2 divisions is virtually pathognomonic of a cavernous sinus lesion (1). The clinical presentation from the case report describes a patient with atypical features of cavernous sinus embolism. Symptoms started with painful ophthalmoplegia without facial sensory impairment. Neurological examination revealed right complete, pupil-sparing third and sixth nerve palsies. It might seem that metastatic tumor, infiltration, carcinomatous meningitis, or paraneoplastic process in the cavernous sinus would be expected in a patient with cancer. However, the brain MRI with contrast did not show any signs of infiltration or a leptomeningeal enhancement. The paraneoplastic anti-neuronal antibody panel of the CSF (including Anti-Hu) was negative, either. Thus, the atypical symptoms and radiological presentations make the diagnosis even more challenging and confusing. The clinical diagnosis was finally verified by the ^18^F-FDG PET scan which revealed FDG accumulation in bilateral cavernous sinuses while the cranial CT scan showed isometric density. After a review of the standardized uptake value (SUV), which is five times greater than the average value of normal tissues, the diagnosis of cavernous sinus cancer embolus was considered.

## Patient's descriptions

A 75-year-old man with a known diagnosis of lung cancer presented with a complaint of recent onset of diplopia and severe headache which appeared 1 week prior. Before the presentation, he had been diagnosed with a non-small-cell lung adenosquamous carcinoma metastasized to the mediastinal lymph nodes 17 months ago. A right upper lobectomy with mediastinal linfadenectomy was done without intercurrences. Based on the TNM international staging system, his lung cancer was classified as T2N3M0. A sequential chemotherapy regimen was initiated, but his last oncology follow-up was 10 months ago.

The sharp right-sided retro-orbital pain was continuous, 8/10 in intensity, and could be partially remitted after vomiting. He denied having fever, photophobia, or phonophobia. The blood pressure was 106/61 mmHg, the pulse was 78 per minute and regular, and the temperature was 36.7 °C. No obvious lung murmur on auscultation was observed. There were no signs of infective focus in the head or elsewhere. Upon neurological examination, the patient was alert and oriented with no speech difficulties. The neck was supple and the carotid pulses were equally palpable with no bruits. He had a right partial upper eyelid ptosis covering 3/4 of the pupil, a right abducents paralysis with 8 mm of sclera showing on right abduction, and an oculomotor nerve dysfunction with reduced supraduction and infraduction, while with no obvious ocular edema or ocular congestion. His pupils were of medium caliber which was about 3 mm, symmetric, and responded to light. The patient was uncooperative, and he refused funduscopic examination. There was no hypoesthesia in the frontal and maxillary regions of his right-side face. Corneal sensation was normal. Other cranial nerve functions were normal. Deep tendon reflexes were hyporactive symmetrically, and motor/sensory, gait, and coordination examinations revealed no obvious abnormality.

What was unexpected was the extremely rapid progression to complete fixation of the right eyeball in 3 days after while pupils were still spared.

**Figure 1:**
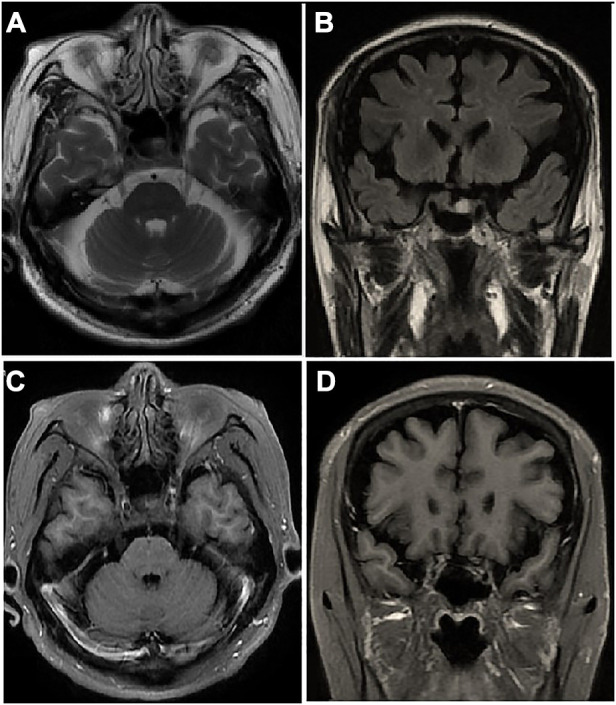
Brain MRI scan of the patient: axial (**A**) and coronal (**B**) images show no microscopic signs of metastases in cavernous sinus. Gadolinium-enhanced axial (**C**) and coronal (**D**) images show no obvious altered dural enhancement along lateral walls of both cavernous sinuses.

Emergency cerebral computerized tomography angiography was normal; therefore, the possibility of an aneurysmal compression was excluded. Brain magnetic resonance imaging (MRI) scan found no microscopic signs of metastases in cavernous sinus (Figures 1A,B) and contrast-enhanced revealed no enhancement of the corresponding cranial nerve (Figures 1C,D). A lumbar puncture revealed 22 cm of cerebrospinal fluid (CSF) opening pressure. A total of 20 mL of CSF was drained, resulting in an 18 cm CSF closing pressure. Fluid analysis revealed a cell count of 0/hpf, a protein count of 601 mg/L, and a glucose count of 62 mg/dl (0.78 of the blood glucose measure). Real-time polymerase chain reaction analysis for common infectious agents of the central nervous system, as well as CSF bacterial, mycobacterial, and fungal cultures were negative, excluding an infectious cause. The paraneoplastic anti-neuronal antibody panel of the CSF (including Anti-Hu) was negative. The cytopathological investigation of the CSF found no malignant cells.

**Figure 2:**
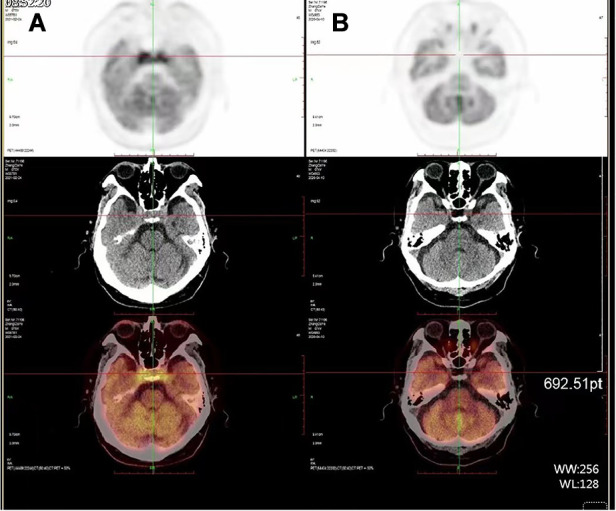
PET-CT scan (**A**) compared with the images 10 months ago (**B**), CT scan shows isointense soft tissue filling the suprasellar region, while FDG accumulation in bilateral cavernous sinuses.

^18^F-fluoro-2-deoxy-d-glucose (FDG)-positron emission tomography (PET)-CT scan revealed accumulation of radioactivity in both lungs and multiple abnormal hypermetabolic lymph nodes in neck, clavicle, mediastinum, and thoracic paravertebral, which were considered metastasis. Compared with the images 10 months ago, there was FDG accumulation in bilateral cavernous sinuses and the CT scan showed isometric soft tissue filling the suprasellar region (Figure 2).

**Figure 3:**
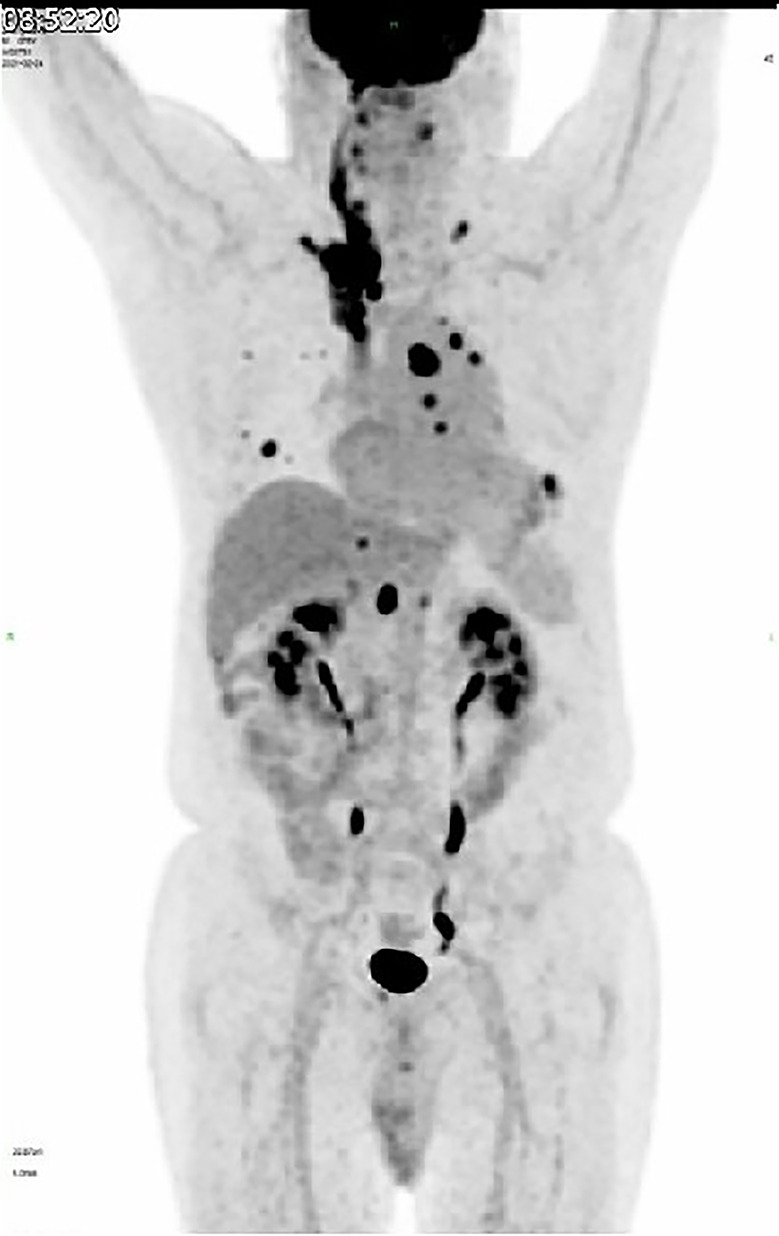
^18^F-FDG-PET scan shows hypermetabolic foci located from the origin of superior vena cava, expanding to the right cephalbrachial vein, with entrance to the right common jugular vein, right internal jugular vein, and vertebral vein.

The hypermetabolic foci were found located from the origin of the superior vena cava, expanding to the right cephalbrachial vein, the right clavicular vein with entrance to the right common jugular vein, the right internal jugular and vertebral veins, and progressed cranially to the suprasellar region (Figure 3). After a review of the standardized uptake value (SUV), which is five times greater than the average value of normal tissues, the diagnosis of venous embolus was considered.

**Figure 4:**
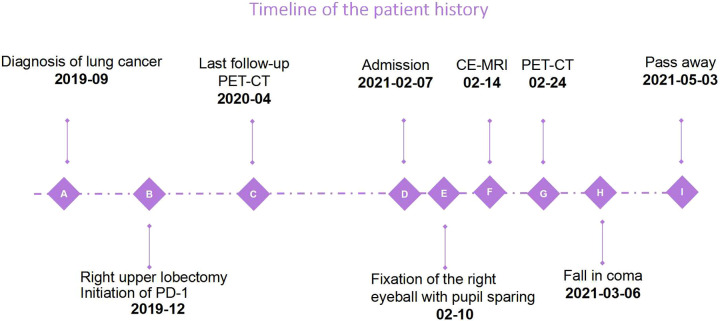
The timeline shows the course of the disease. Due to the palliative regimen, the patient deteriorated rapidly and passed away 3 months after admission.

Based on the poor prognosis, the patient opted for palliative care and fell into a coma about a month after admission, and eventually passed away 2 months later (Figure 4).

## Discussion

We report a case of ^18^F-FDG-PET confirmed cavernous sinus cancer embolus in an old patient with advanced lung cancer, which was rare and life-threatening.

Lung cancer is the leading cause of death worldwide (2). Beninato et al. (3) reported that lung cancer is associated with a particularly high risk of venous thromboembolism. In the literature review, we found cavernous sinus embolism is almost invariably septic and is usually associated with a primary infective source in the face, throat, mouth, ear, mastoid, or in the sphenoidal, ethmoidal, or maxillary sinuses (4–7). Aseptic cavernous sinus embolism is a rare occurrence and has been described to be associated with head injury, surgical procedures of the fifth nerve, and carcinomatous invasion of the base of the skull (8–10). To our knowledge, venous tumor thrombus progressed cranially to the suprasellar region with painful ophthalmoplegia as the first manifestation of aseptic cavernous sinus embolism has not been previously reported.

While more clearly described in the setting of infection wherein localized inflammatory processes related to septic emboli, thrombi formation, and localized infection are thought to drive thrombus formation (11), the pathogenesis of cavernous sinus embolism in the setting of malignancy is not well-defined. In the absence of tumor compression or invasion, malignancy-related prothrombotic states are thought to drive the development of emboli *via* a variety of mechanisms including a tumor's ability to increase the expression of tissue factor, cancer procoagulant, and inflammatory cytokines as well as to downregulate the expression of thrombomodulin and the protein C system (12). Therefore, it is reasonable to assume that the elements comprising Virchow's triad (vascular stasis, endothelial damage, and hypercoagulability state secondary to the malignancy) play a vital role in the pathogenetic mechanism underlying this unusual syndrome.

Although headache and double vision are reported as common ophthalmic symptoms with cavernous sinus embolism (13), complete pupil-sparing painful ophthalmoplegia without chemosis or periorbital edema is unusual. Compressive lesion causing a CN III palsy is usually manifested as pupillary dilation, as the parasympathetic fibers of the oculomotor nerve are distributed in the nerve surface, while the central part of the nourishing artery lesions leads to pupil-sparing oculomotor palsy. If it was tumor direct compression or encroachment upon adjacent cranial nerves, CN III involvement with pupil dilation would be expected, but since it was not involved, alternative mechanisms might have existed. The specific honeycomb-like structure of the cavernous sinus might be associated with this uncommon nerve palsy manifestation.

Cavernous sinus embolism is a potentially fatal neurological condition that is often under-diagnosed due to its nonspecific clinical and radiological presentation. Anticoagulation with heparin or low-molecular-weight heparin is the mainstay of treatment. Endovascular management is indicated for those cases with severe symptoms or worsening of symptoms despite anticoagulation therapy. Favorable outcomes have been reported in patients who receive early diagnosis and treatment. This patient was advised of anticoagulant therapy after the diagnosis was made, but he refused due to the risk of bleeding. Increasing attention should be paid to the importance of embolism in cancer patients. Early identification and early diagnosis can effectively reduce morbidity and mortality.

The diagnosis of cavernous sinus embolism remains a challenge despite advancements in radio-imaging technologies (14). Amongst the various venous connections of the cavernous sinus, the superior and inferior ophthalmic veins are important tributaries that drain into the cavernous sinus. When there is an embolus in the sinus, there is impaired drainage of blood from the superior ophthalmic vein, thus leading to dilatation and/or subsequent thrombosis. According to the literature (15), a cut-off value of SOV diameter of ≥2.9 mm was considered to be 100% sensitive, specific and accurate in predicting cavernous sinus embolism. In our patient, no dilated SOV was observed. It might be more specific when combined with other parameters such as the diameter of the sinus and was more often seen in patients with septic causes who presented with ocular edema and ocular congestion.

Cavernous sinus, being a separated venous space containing flowing blood, produces negligible signal intensity on plain MRI sequences (16). On post-contrast images, the inflammatory soft tissue unusually showed heterogenous enhancement (17), whereas, due to aseptic causes, the patient did not show any altered signal intensity on contrast-enhanced brain MRI. ^18^F-FDG-PET has proven to be a promising indicator of cancer embolus in patients with chest malignancies.

## Conclusion

Unilateral oculomotor nerve involvement with pupil-sparing heralding the cavernous sinus embolism is rare. Cavernous sinus cancer embolism has high morbidity and mortality. In summary, our case suggests that if the symptoms progressed quickly in lung cancer patients tentatively diagnosed with cavernous sinus syndrome, the possibility of cancer embolus should be considered. Early identification and early anticoagulant therapy might significantly improve the clinical prognosis of patients. The MRI has insufficient sensitivity in the diagnosis of cancer embolus, whereas the ^18^F-FDG-PET scan has obvious advantages in diagnostic sensitivity and specificity.

## Data Availability

The original contributions presented in the study are included in the article/Supplementary Material, further inquiries can be directed to the corresponding author/s.
